# O-Linked β-*N*-Acetylglucosamine Modification: Linking Hypertension and the Immune System

**DOI:** 10.3389/fimmu.2022.852115

**Published:** 2022-03-17

**Authors:** Rinaldo Rodrigues dos Passos Junior, Gisele Facholi Bomfim, Fernanda R. Giachini, Rita C. Tostes, Victor Vitorino Lima

**Affiliations:** ^1^ Institute of Biological and Health Sciences, Federal University of Mato Grosso, Barra do Garças, Brazil; ^2^ Institute of Biological Sciences, Federal University of Goias, Goiânia, Brazil; ^3^ Institute of Health Sciences, Federal University of Mato Grosso, Sinop, Brazil; ^4^ Department of Pharmacology, Ribeirão Preto Medical School, University of São Paulo, Ribeirão Preto, Brazil

**Keywords:** O-glcnacylation modification, immune system (IS), hypertension, adaptive immunity (ADIM), innate immunity (basic sciences)

## Abstract

The *O*-linked β-*N*-acetylglucosamine modification (O-GlcNAcylation) of proteins dynamically regulates protein function, localization, stability, and interactions. This post-translational modification is intimately linked to cardiovascular disease, including hypertension. An increasing number of studies suggest that components of innate and adaptive immunity, active players in the pathophysiology of hypertension, are targets for O-GlcNAcylation. In this review, we highlight the potential roles of O-GlcNAcylation in the immune system and discuss how those immune targets of O-GlcNAcylation may contribute to arterial hypertension.

## Stating the Problem

O-GlcNAcylation on serine (Ser), threonine (Thr) and tyrosine (Tyr) residues of nuclear, cytosolic, and mitochondrial proteins is one of the most abundant post-translational modifications that modulate phosphorylation, stability, and activity of multiple cellular signaling pathways and transcription regulatory cascades (1, 2).

**Figure 1:**
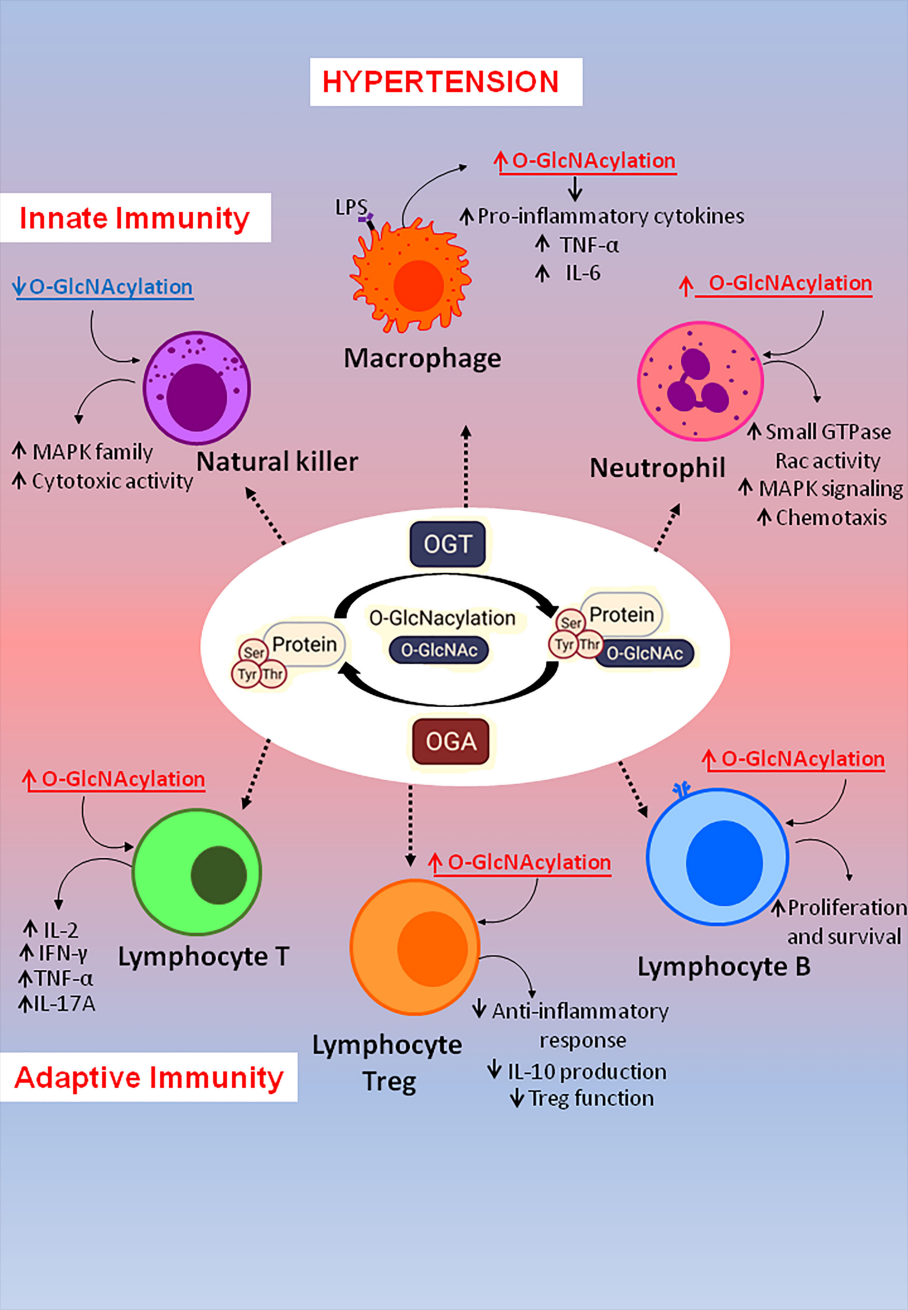
Factors linked to hypertension also related to O-GlcNAcylation levels. Hypertension is a multifactorial disease involving many factors and events, which are also associated with excessive chronic O-GlcNAcylation levels.

O-GlcNAc is dynamically, reversibly, and rapidly cycled on and off proteins by two specific enzymes: O-GlcNAc transferase (OGT), which catalyzes the addition of the O-GlcNAc moiety; and O-GlcNAcase (OGA), that catalyzes its removal (**Figure 1**). This modification is responsive to several stimuli, including nutrient availability. An overabundance of nutrients can drastically shift substrates to the hexosamine biosynthetic pathway (HBP), favoring the synthesis of the O-GlcNAc precursor, UDP-GlcNAc, manly *via* recruitment of the rate-limiting enzyme glutamine:fructose-6-phosphate aminotransferase (GFAT) (2, 3). However, many studies show that protein O-GlcNAcylation excess occurs in response to factors that do not fit neatly with the nutrient availability concept, including oxidative stress (4, 5), renin-angiotensin system (RAS) (6–8) and endothelin-1 (ET-1) (9–12).

O-GlcNAc-modified proteins have been implicated in a diverse array of cellular functions, including signaling, transcription, apoptosis, and inflammation (2, 13). Given its diverse roles, protein O-GlcNAcylation has been associated with a diverse array of pathological conditions, such as arterial hypertension.

Hypertension affects over 1.2 billion individuals worldwide and is a multifactorial disease involving immune cells activation, inflammation, oxidative stress, activation of the sympathetic and renin angiotensin aldosterone (RAAS) systems, and others. This might explain the fact that the etiology of hypertension is known in only 10% of the patients (14). Many therapeutical classes of drugs targeting many different molecules are available to treat hypertension based on the classical view of blood pressure (BP) control by neural, vascular and renal mechanisms. Still, 20% of hypertensive patients do not adequately achieve BP control (15). With this refractory response to the regular anti-hypertensive therapeutic arsenal in mind, scientific advances have been targeting the immune system, as a possible therapeutic target to treat hypertension (16).

The present review will provide a brief overview of the scientific advances related to the role of O-GlcNAcylation on hypertension progression. Then, the focus will be to set on potential mechanisms whereby O-GlcNAcylation of components of the immune system could impact hypertension.

## O-GlcNAcylation and Arterial Hypertension

It is well-recognized that hyperglycemia, linked to the diabetic condition, negatively impacts cardiovascular function and, consequently, blood pressure control (17). Yet, little is known regarding how augmented flux of glucose, or other subproducts from the nucleotide and fatty acid metabolism, through the hexosamine biosynthetic pathway (HBP) contributes to the pathophysiology of hypertension.

The HBP is highly sensitive to the major metabolites produced in glucose, amino acid, nucleotide, and fatty acid metabolism. Therefore, an overabundance of nutrients (18), as seen in diabetic or hyperlipidemic subjects, may overload the HBP flux, leading to increased protein O-GlcNAcylation (19). In addition to diabetes, increased O-GlcNAc levels have been reported in different experimental models of hypertension (6, 20, 21), cardiac hypertrophy (22, 23), cardiac dysfunction (22, 24, 25), as well as in response to agonists such as angiotensin II (Ang II) (6) and endothelin-1 (ET-1) (9, 10). The fact that O-GlcNAc impacts biological functions that are not totally linked to altered nutrient availability, raised the suggestion that other mechanisms regulating O-GlcNAcylation may participate in the pathophysiology of hypertension.

Increases in arterial blood pressure can be generated by a variety of events, including modifications in cardiac output, vascular dysfunction, and target organ damage (14, 17). Interestingly, well-known mechanisms whereby blood pressure (BP) and cardiac function may be altered are also targeted by O-GlcNAcylation (**Figure 1**).

Excessive chronic O-GlcNAcylation has been shown both in humans and animal models of myocardial dysfunction, cardiac remodeling, aortic banding, and hypertension (22, 25–27). Hyperglycemia in Zucker (diabetic fatty) rats leads to high O-GlcNAc levels along with attenuated cardiomyocytes calcium peak and prolonged time to relaxation and, consequently, to impaired cardiac contraction and relaxation in these animals (28).

Chronic increases in O-GlcNAc levels also leads to increased risk of ventricular arrhythmias, which has been linked to increased O-GlcNAcylation of cardiac voltage-gated sodium channels (26).

Cardiac hypertrophy is usually seen in different stages of hypertension (29, 30). In the long-term, cardiac O-GlcNAcylation is also increased in cardiac hypertrophy conditions (22, 31). High levels of O‐GlcNAc are seen in hypertrophic hearts in response to phenylephrine (32, 33). However, hypertrophy was not observed in cardiomyocytes treated with an inhibitor of GFAT (23), a rate-limiting enzyme in the HBP pathway. These results suggest that high levels of O-GlcNAc are associated with cardiac hypertrophy and inhibition of the O-GlcNAc pathway may work as a pharmacological tool to block hypertrophy progression during hypertension.

Considering the evidence linking the O-GlcNAc involvement with ventricular arrhythmias, cardiac hypertrophy and other cardiac dysfunctions, it is reasonable that O-GlcNAc may influence important factors related to hypertension (24). In this sense, experimental models of hypertension, such as spontaneously hypertensive rats (SHR), have significantly higher systolic blood pressure, hypertrophy and increased protein O-GlcNAcylation in the left ventricle (LV). This study showed that increased O-GlcNAc in pressure overload conditions, including chronic hypertension and aortic banding, is associated with increased OGT protein, suggesting that OGT levels might be an important mechanism for cardiac protein O-GlcNAcylation. Of importance, O-GlcNAc levels are 65% higher in LV biopsies from patients with severe aortic stenosis, compared with controls (22).

Furthermore, a correlation between increased O-GlcNAcylation and GFAT expression was reported in SHR (20, 22). Silva-Aguiar et al. showed that adult SHR with established hypertension display increased renal cortical O-GlcNAcylation. They proposed that changes in protein located at the proximal tubule are associated with an increase in O-GlcNAcylation in the renal cortex of adult SHR. Conversely, no changes in O-GlcNAc levels or blood pressure were observed in young SHR, suggesting that increased cortical O-GlcNAcylation could be related to the development of hypertension. In agreement, a GFAT inhibitor reduced global O-GlcNAcylation and also significantly decreased blood pressure in SHRs (20).

ET-1, a potent vasoconstrictor peptide and growth-promoting factor (34), also plays an important role in the physiological control of blood pressure and in the genesis and development of arterial (35) and pulmonary hypertension (36). ET-1 levels are increased in the vasculature of deoxycorticosterone acetate (DOCA)-salt hypertensive rats (Schiffrin, 2005). Moreover, increased vascular GFAT expression and O-GlcNAcylation was correlated with time-dependent increases in blood pressure and vascular dysfunction in DOCA-salt hypertensive rats (21). In agreement, *in vitro* and *in vivo* treatment with ET-1 increases vasoconstriction and the vascular content of O-GlcNAc-modified proteins. Interestingly, these effects were not observed when vessels were previously transfected with antibodies against OGT or incubated with an OGT inhibitor (10).

Chronically increased BP occurring similarly to augmented O-GlcNAc tissular levels may be an additional mechanism eliciting end-organ damage (20). For example, chronically increased O-GlcNAcylation was positively correlated with renal damage (12, 37), and patients with nephropathy display increased glomerular and tubular O-GlcNAcylation (38). Moreover, a number of studies also provide evidence for an interplay between protein O-GlcNAcylation and Ang II, a well-known vasoactive that increases blood pressure (29), induces cardiac hypertrophy (39) and kidney damage (40, 41). Ang II increases O-GlcNAcylation in mesangial cells (6) and heart from mice (7). Conversely, Ang 1–7 and Mas-receptor inhibition reduced protein O-GlcNAcylation by repressing GFAT1 activity (8).

Gellai et al. showed that RAAS inhibitors inhibit diabetes-induced O-GlcNAcylation in the kidney, by increasing OGA expression (12). In line with this view, changes of protein O-GlcNAcylation levels resulted in a concomitant alteration in angiotensinogen, OGT, and GFAT transcripts (42). Furthermore, *in vivo* glucosamine-treatment increased the expression of angiotensinogen in adipose tissue (43). Therefore, O-GlcNAcylation regulates local and systemic RAAS, which may contribute to the progression of hypertension. Finally, high levels of O-GlcNAcylation driven by high glucose or glucosamine treatment leads to impaired vascular endothelial and smooth muscle cells function in human and rat (11, 25, 44–46), a phenotype closely associated with hypertension (29). In this sense, there is direct evidence that elevated levels of protein O-GlcNAcylation in endothelial cells impairs endothelium-dependent relaxation (44, 47–49), demonstrating that O-GlcNAcylation leads to endothelial dysfunction. Furthermore, O-GlcNAcylation of vascular smooth muscle cells augments vascular response to contractile agonists (50, 51) and favors vascular calcification (52–54), resulting in high blood pressure (25).

## Possible Mechanisms Whereby the Crosstalk Between O-GlcNAcylation and the Immune System Impacts Arterial Hypertension

**Figure 2:**
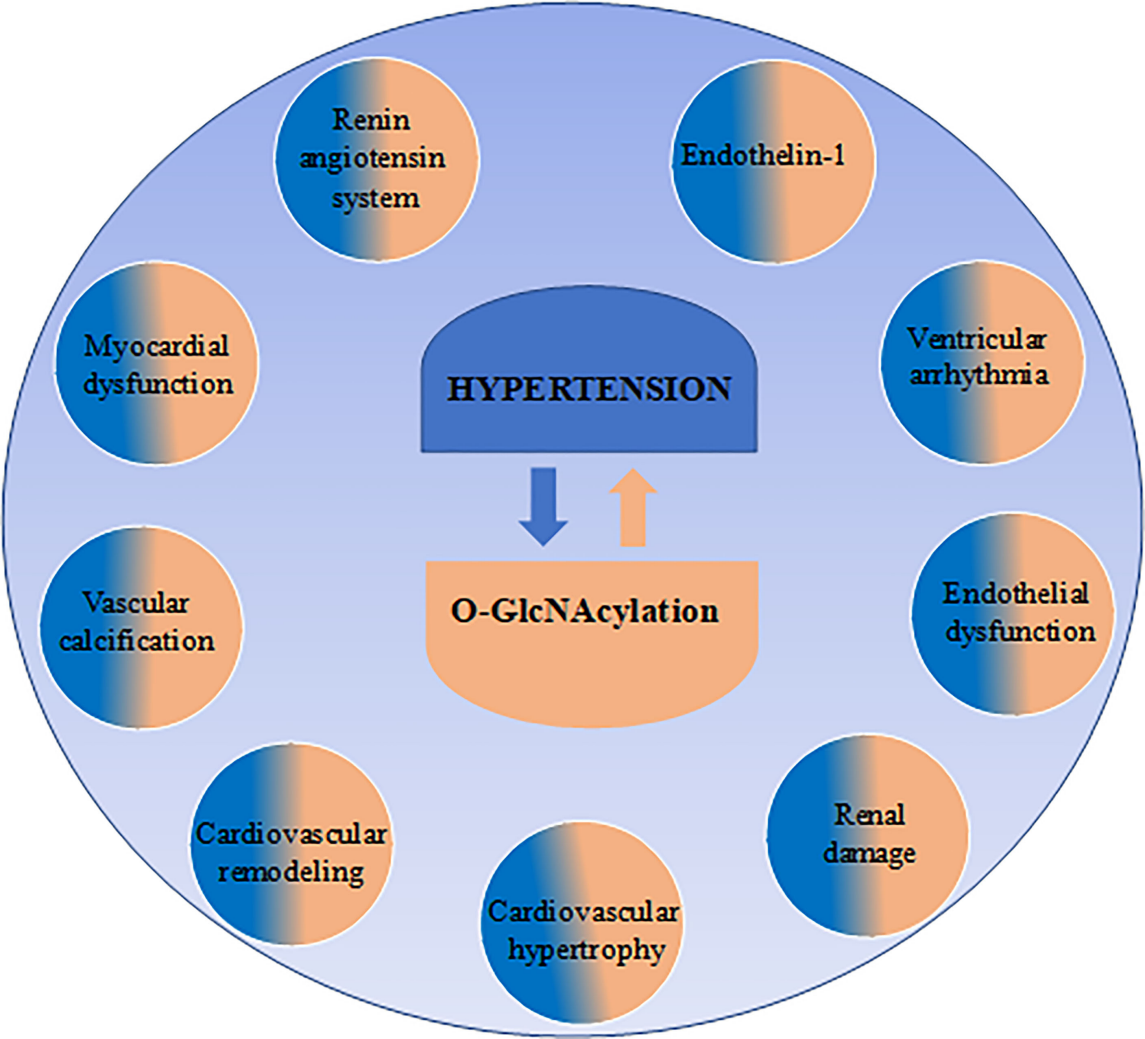
Immune targets of O-GlcNAcylation may contribute to arterial hypertension. O-GlcNAcylation is a dynamic and reversible modification in protein residues by the actions of two specific enzymes: O-GlcNAc transferase (OGT) and O-GlcNAcase (OGA). Regarding innate immunity: O-GlcNAc plays important roles in macrophages and neutrophils by favoring pro-inflammatory cytokine production, neutrophil infiltration and motility, which are associated with the severity of hypertension. Additionally, in natural killer (NK)-cells, reduced O-GlcNAc modification was found to favor NK cell cytotoxicity by enhancing MAPK family activity. Regarding adaptive immunity: O-GlcNAcylation was found to target T cells, T reg cells and B cells. In lymphocytes T, global elevation of O-GlcNAc modification favors IL-2 production and cell proliferation, as well as Th1 pro-inflammatory IL-17A and IFNγ cytokine secretion, which are associated with hypertension. In lymphocyte Treg was found that O-GlcNAc actions impaired anti-inflammatory response through STAT-3 O-GlcNAcylation, affecting STAT3–IL-10 axis and impairing Treg function. Additionally, in B cells, protein O-GlcNAcylation enhances B cell proliferation and survival. Interestingly, B cells were found to be related to increased blood pressure.

Classically, the main function of the immune system is to defend a host against pathogen invasion. However, as extensively reviewed in the literature, overactivation of immune system components contributes to non-infectious disease, like hypertension (16, 55, 56). The immune response is complex and has two interconnected systems: the innate immunity, which mediates early reactions, and adaptive immunity, which is a late and more specific response. Both systems are activated and contribute to high blood pressure and tissue damage in hypertension. The interplay between O-GlcNAcylation and the immune system has gained great interest in the last few years when several studies have been performed, highlighting how this post-translational modification impacts the immune cells. In this chapter we will highlight potential mechanisms whereby O-GlcNAcylation of immune components may contribute to hypertension (**Figure 2**).

### Involvement of Innate Immunity in Hypertension: Possible Roles of O-GlcNAcylation

The innate immune response is initiated by the recognition of antigens originated from debris and other molecules from damaged cells, known as DAMPs (damage associated molecular patterns); or alternatively, from antigens originated from pathogens, called PAMPs (pathogen associated molecular patterns) (57). DAMPs and PAMPs are recognized by pattern recognition receptors (PRR), including Toll-like receptors (TLR) family, which are expressed in several cells.

There are 11 subtypes of TLRs, and the participation of TLR2, TLR3, TLR4, TLR7, and TLR9 receptors in hypertension has been described (58, 59). Activation of TLR4 is directly involved on vascular inflammation, vascular dysfunction, and hypertension (60, 61). Vascular TLR4 expression is increased in cardiovascular disease, including several animal models of hypertension (SHR, Ang II infusion, DOCA-Salt), atherosclerosis and others (18, 62). Treatment with a neutralizing anti-TLR4 antibody decreases blood pressure, vascular inflammation, and cardiac remodeling in hypertensive rats (60, 61, 63). TLR4 overexpression aggravates vascular smooth muscle cells proliferation and vascular remodeling in hypertension (64). TLR9 stimulation produces systemic maternal inflammation and vascular dysfunction that lead to hypertension (65). A TLR9 antagonist decreased blood pressure in SHR and TLR9 agonist impairs mesenteric resistance arteries’ function and increases local ROS (66). Mitochondrial DNA is a DAMP that activate TLR9. Echem et al. (63) demonstrated that only male SHR exhibit high levels of plasma mitDNA and the antagonism of TLR9 normalizes mitDNA-induced increased aortic contractions elicited to phenylephrine (67). TLR9 also negatively modulates cardiac vagal tone and baroreflex in mice (68).

After the PRR recognize specific antigens, innate immunity cells are activated and contribute to the inflammatory profile in hypertension. Circulating and endothelial cell adhering monocytes are increased in many hypertensive animal models and hypertensive patients (69–74). Monocytes isolated from hypertensive patients are pre-activated and secrete high levels of IL-1β after Ang II stimulation (75). Of importance, macrophage infiltration is recognized as a classical histological characteristic of end-organ injury in hypertension (70, 76).

As previously mentioned, TLRs are known to initiate innate immune response in several cells, such as macrophages, neutrophils, natural killer (NK), dendritic cells, and mast cells (77). Thus, regulation of TLRs activation is a critical step to ensure adequate immune responses.

Macrophages can switch to a distinct functional phenotype in response to physiological and microenvironmental signals and stimuli. The classically activated M1 macrophages are known to possess a pro-inflammatory phenotype, secreting pro-inflammatory cytokines, aiming to kill pathogens. They may be characterized by TLR-2, TLR-4, CD80, CD86, iNOS, and MHC-II surface phenotypes, secreting pro-inflammatory cytokines and chemokines, promoting cell proliferation and tissue repair (78, 79). Beyond involvement in the innate immune response, there is direct evidence that macrophages *per se* may affect blood pressure. For example, patients with hypertension display macrophage infiltration into the vascular wall, myocardium and kidneys (80). Moreover, Ang II-induced increased blood pressure is attenuated in macrophages-deficient mice, showing the contribution of these cells to hypertension (81).

When macrophages are activated by LPS/TLR4, M1 macrophage polarization is induced, resulting in reduced HBP activity and attenuated protein O-GlcNAcylation (82). However, increased O-GlcNAcylation in LPS-stimulated macrophages, or after intraperitoneal injection of LPS in mice have also been reported (83). In this case, when macrophages are activated by LPS, OGT is activated, enhancing protein O-GlcNAcylation, which in turn favors cytokine production, and a pro-inflammatory environment.

Among the pro-inflammatory cytokines produced by M1 macrophage and other immune cells, tumor necrosis factor alfa (TNF-α) and interleukin (IL)-6 are crucial to this inflammatory profile. Therefore, NF-κB activation is a key component of the stimulation of innate immunity by PRR recognition of PAMPs/DAMPs, inducing the production of pro-inflammatory cytokines, which plays an essential role in the hypertension (84). Interesting, O-GlcNAcylation was found to modulate this transcription factor (85). NF-κB transcriptional activity is induced by OGT, and OGT colocalizes with promoter regions of NF-κB, enhancing RelA acetylation upon TNF-α stimulus, as observed in human embryonic kidney (HEK 293) cells (86). c-Rel, one of the five NF-κB subunits, is a target for O-GlcNAcylation at serine350, a mandatory post-translational modification required for c-Rel binding to the DNA, allowing transcription, as demonstrated by RAMOS in a B lymphocyte line and in Jurkat cells (immortalized line of human T lymphocytes (87). LPS also favors O-GlcNAcylation of c-Rel in the iNOS promoter in BV2 microglia cells (88). In RAW 264.7 cells, a cell line of mouse macrophages, overexpression of OGT inhibited NF-κB reporter activity upon LPS stimulus, resulting in NF-κB/iNOS transcription suppression (89). Therefore, these data on macrophages indicate that immune dysfunction may be elicited both by augmented and diminished protein O-GlcNAcylation.

Neutrophil lymphocyte ratio is an inflammatory marker, which has strong independent association with the severity of hypertension (90–93). During inflammation, neutrophils are the first cells to arrive at the injured site, ready to orchestrate the repair of tissue damage induced by macrophages. Neutrophils are polymorphonuclear leukocytes that enter the circulation and migrate towards other tissues, are responsible for patrolling the organism and searching for pathogens and other signs of infection (94). To reach sites of injury, neutrophils migrate under the regulation of intracellular signaling in a mechanism called chemotaxis. These signaling pathways release molecules that stimulate neutrophils to migrate towards the injury site (95). One of the most commonly used polymorphonuclear leukocytes stimuli, the chemotactic tripeptide formyl-methionine-leucine-phenylalanine (fMLF), binds to cell surface receptors and induces protein phosphorylation within seconds.

O-GlcNAc-modified proteins are increased in polymorphonuclear leukocytes stimulated with fMLF-stimulus, and glucosamine increases O-GlcNAc and enhances neutrophils motility (96). In addition, pharmacological increases in O-GlcNAc, using glucosamine or PUGNAc (OGA inhibitor), increases the activity of the small GTPase Rac and MAPK signaling in neutrophils (97). These protein kinases are involved in neutrophil chemotaxis and Rac is known to activate MAPK (98). This evidence shows that O-GlcNAcylation is required for neutrophil signaling transduction, including chemotaxis. In fact, augmented O-GlcNAcylation evokes both neutrophil chemotaxis and mobility (96, 97). Importantly, the downregulation of the immune system appears as a key factor in the treatment of hypertension. As a proof of this concept, a selective antagonist of β1-adrenoceptors, nebivolol, efficiently reduced innate immune responses in hypertensive patients, through decreased levels of neutrophils (99). Therefore, the role of O-GlcNAc during neutrophil chemotaxis and mobility may be a predictor of ongoing vascular inflammation in various cardiovascular disorders such as hypertension.

NK cells are a large granular type of cytotoxic lymphocytes that are essential for innate immune response, acting rapidly against a great number of pathogens and constantly interacting with other immune cells, such as macrophages and dendritic cells (100). Compared to normotensive rats, SHRs have a strong increase in the number of NK cells (101). Depletion of NK cells, using an anti-NK antibody, protects against Ang II-induced vascular dysfunction (102). Furthermore, an association between increased proportions of NK-cells and levels of systolic blood pressure has been detected in a large multi-ethnic cohort (103).

NK cells recognize infected cells through activation of receptors such as natural killer group 2D (NKG2D), which require the actions of the transcription factor enhancer of zeste homolog 2 (EZH2) (104, 105). Interestingly, few studies found that EZH2 O-GlcNAcylation is required for EZH2 protein stability and enzymatic function in human breast and colorectal cancer cells (78, 79, 106). Upon activation through its receptors, NK cells exert their cytotoxic activity killing aberrant cells, such as infected and tumorigenic cells, through the release of cytotoxic molecules stored in exocytic organelles (107). The cytotoxic vesicles release depends on MAPK family and extracellular signal-regulated kinases (ERK) activation (108). ERK-2 MAPK-dependent pathway becomes activated and mediates the movement of intracellular granules (109). A study reported that protein O-GlcNAcylation may be involved in the cytotoxic signal transduction of NK cells. There is reduced O-GlcNAc modification during NK cell cytotoxicity and inhibiting NK cytotoxicity by GST-sHLA-G1α chain restores O-GlcNAcylation in NK92 cells (110). Moreover, glucosamine treatment exerts an inhibitory effect on NK-92 cell cytotoxic vesicles release by increasing O-GlcNAc modification of ERK downstream proteins, increasing ERK nuclear localization and altering granules migration (111). The same study also showed that after, glucosamine treatment, the transcription factor FOXO1 presented reduced phosphorylation and increased O-GlcNAcylation. Interestingly, FOXO1 was found to negatively regulate NK cells’ function (112). Altogether, these data elucidate the role of protein O-GlcNAcylation on NK cell function.

The wide range of responses elicited by O-GlcNAcylation in innate immune cells indicates an important, but still incomplete unknown, role for this post-translational modification on inmate responses.

### Involvement of Adaptive Immunity in Hypertension: Possible Roles of O-GlcNAcylation

Adaptive immunity, also referred to as acquired or specific immunity, is the second and long-lasting line of the host’s defense against non-self particles or pathogens. The main characteristic of the adaptive immune response is the clonal expansion of lymphocytes, such as T and B lymphocytes. There are two primary subtypes of T cells: cytotoxic T cells (CTLs), and helper T cells (Th). Experimental and clinical studies have shown the importance of adaptive immune system in hypertension (113). The main cells subtypes that contribute to hypertension are Th1, Th17, regulatory T cells (Treg), T CD8 and B cell.

#### Possible Target: T Cells

The association of T lymphocytes with hypertension has been supported by many studies showing that mice lacking T- and B-lymphocytes exhibit attenuated hypertension in response to Ang II (114–117).

Th1, Th2, Th17 and Treg are subtypes of CD4 T cells. Th1 cells express the transcription factor T-bet and release the cytokine IFN-γ. T-bet deficient mice infused with Ang II is protected against renal injury, but not from high blood pressure (118). Isolated T cells from the spleen of Ang II-infused rats exhibit an imbalance of Th1/Th2 subsets, with increased IFN-γ (Th1 cytokine) and decreased IL-4 (Th2 cytokine) (119). IFN-γ knockout mice are protected from Ang II-induced vascular and kidney dysfunction (102, 120). Although some studies show the contribution of IFN-γ in hypertension, other cell subtypes like LT CD8 and NK-T can also release this cytokine. Therefore, the association between the activation of the immune system and hypertension is clear.

The role of O-GlcNAcylation, as well as OGT, on T cells activation has been investigated before (121–123). One of the earliest studies in this field demonstrated that murine T lymphocytes activation resulted in decreased levels of O-GlcNAc-modified cytosolic proteins with a concomitant increase of this post-translational modification in the nucleus, establishing the role for protein O-GlcNAcylation in the early stages of T-cell activation (123). This was further supported by a study that found that blocking O-GlcNAc cycling disrupts early T cell development in mice (124).

The nuclear factor of activated T cells (NFAT) allows transcriptional induction and release of IL-2, as well as other cytokines such as IL-4, IFNγ, and TNFα, as observed in activated T cells (125). NFAT is a target for O-GlcNAc and silencing OGT through siRNA-mediated knockdown, impairs activation of NFAT and NFκB, reducing IL-2 production, and consequently preventing T cell receptors (TCR)-induced activation (121). Thus, OGT is required for T and B cell activation. These data was further supported by Lund et al. (126), who found that activation of T cells through the TCR resulted in a global elevation of O-GlcNAc levels. Yet, in the absence of O-GlcNAc, IL-2 production and T cell proliferation were compromised (126). Controversially, augmented O-GlcNAc levels in heart tissue and rat cardiomyocyte-derived cell line suppressed NFAT and NF-κB activity through GSK-3β protein O-GlcNAcylation (127). It seems that O-GlcNAcylation antagonizes NFAT effects, since GSK-3β is known to negatively regulate NFAT activity (128). Collectively, these data show that this post-translational modification is crucial for T cell activation, whereas aberrant protein O-GlcNAcylation may be deleterious.

Th17 and γδ T cell release IL-17, which is associated with hypertension. Kim et al. (129) demonstrated that transfer of Th17 cells from adult SHR accelerates the development of hypertension in juvenile SHR (129). Ang II infusion increases IL‐17A production by T cells and IL‐17 protein in the aortic media and the heart (130). Hypertension is not sustained in IL-17A^-/-^ mice infused with Ang II. These mice are also protected against aortic stiffening and cardiac fibrosis (130, 131).

Regarding O-GlcNAcylation evoking immune response through T cell, Ramakrishnan et al. (87) demonstrated that hyperglycemia promoted NF-κB and c-Rel O-GlcNAcylation, promoting autoimmunity through enhancing the release of cytokines by helper Th cells (87). Elevated O-GlcNAc levels, through OGA inhibitor, correlate with increased Th17 and Th1 pro-inflammatory IL-17A and IFNγ cytokines secretion by murine and human CD4+ T cells (132). Additionally, Liu et al. (133) found that the microRNA-15b contributes to multiple sclerosis by negatively regulating Th17 cell differentiation, by targeting the OGT enzyme (133). Moreover, microRNA-15b suppressed retinoic acid-related orphan receptor (ROR)γT activation by diminishing NFκB protein O-GlcNAcylation. These findings provide evidence of the importance of O-GlcNAcylation in CD4+ T cells differentiation, since (ROR)γT is known to play a key role in the differentiation of Th17 cell lineage.

#### Possible Target: Regulatory T Cells (Treg)

Another specialized subpopulation of CD4+ T cells is the Treg. These cells play a key role in maintaining the homeostasis of the immune system, regulating the balance between pro-inflammatory and anti-inflammatory responses, and preventing autoimmune responses

In SHR, peripheral blood and splenic Treg cells are markedly diminished, whereas Th17 cells are enhanced (134). In fact, Treg have a protective effect in hypertension. A clinical study demonstrated that during hypertension, end organ damage and arterial stiffness in children is associated with decreased population of circulating Treg (135). Adoptive transfer of Treg prevents Ang II–induced hypertension, vascular damage, and vascular immune cell infiltration (114). Treg adoptive transfer also prevents kidney macrophages infiltration, vascular dysfunction and vascular oxidative stress induced by aldosterone (136).

Treg cells express high levels of IL-2 receptor α (IL-2Rα) chain in its surface and the forkhead box protein P3 (FOXP3) transcription factor in the nucleus, which are important for Treg function and cell fate (137, 138). O-GlcNAc-deficient mice due Treg cell-specific deletion of OGT, display reduced FOXP3 expression, impaired Treg function, and aggressive and lethal autoimmunity (139). Furthermore, deficiency in protein O-GlcNAcylation results in attenuation of IL-2/STAT5 activity in Treg cells. IL-2R activity is known to depend on STAT5 to regulate FOXP3 expression and promote Treg development, thus IL-2/STAT5 is required for FOXP3-induced differentiation of Treg (140). The same importance can be given to Th2 cells, once STAT5 activation is also crucial for Th2 differentiation (141).

IL-10 (STAT3/IL-10) is an anti-inflammatory cytokine released by Treg and other immune cells. IL-10 knockout mice infused with Ang II exhibit greater vascular contractions and IL-10 infusion prevents blood pressure increase in Ang II-infused mice (142). O-GlcNAcylation of signal transducer and activator of the transcription (STAT)-3 resulted in defective STAT3 phosphorylation and IL-10 production, affecting STAT3–IL-10 signaling in macrophages, increasing disease severity in colitis models while inhibition of OGT-mediated O-GlcNAcylation protects against intestinal inflammation (143). The STAT3–IL-10 axis is essential for an anti-inflammatory response, since STAT3 is a key transcriptional factor mediating IL-10 production (144, 145). Thus, STAT-3 protein O-GlcNAcylation impairs its activation, affecting STAT3–IL-10 anti-inflammatory responses. This is further supported by the fact that elevated protein O-GlcNAcylation enhances innate immune responses by increasing NF-κB signaling, and by counterbalancing the anti-inflammatory STAT3-IL-10 signaling in macrophages (82).

#### Possible Target: Cytotoxic T Cells

Once activated through TCR stimulation, T cells differentiate into cytotoxic effector cells (CD8+) and undergo clonal expansion and maturation to become activated CD8+ T cells (146). In addition to CD4 T cells, CD8 cells are also activated and increased in hypertension. Trott et al. (2014) reported that an oligoclonal population of CD8+ cells accumulate in the kidney and contribute to sodium retention and volume expansion, and vascular rarefaction during hypertension development (147). Resistant hypertension patients submitted to renal denervation and with blood pressure control show low values of CD4, CD8 and naïve CD8 T cells, leanding to the suggestion that T cells can be cellular biomarkers that predict the response to renal denervation (148).

CD8 T cells travel through the blood towards tissues looking for cells infected by pathogens, and inducing cell lysis and apoptosis in order to resolve the infection (149). This process involves many post-translational modifications such as phosphorylation and O-GlcNAcylation. Interestingly, protein O-GlcNAcylation is strongly involved in both transcriptional and translational processes that prompt T cells formation and proliferation, in effector-like T cells and memory-like T cells, establishing the role for O-GlcNAc in CD8+ T cells function (150).

#### Possible Target: B Cells

B cells are bone marrow-derived cells that play crucial functions in adaptive immunity, such as antibody production, antigen presentation, and cytokine production and release (151). In the bone marrow, progenitor B cells undergo pro-B, early pre-B, and late pre-B stages to become immature B cells (152). B cells also contribute to Ang II hypertension. B cells genetically deficient mice infused with Ang II present lower blood pressure and B cell transfer rescues this response. Knockout mice are also protected from Ang II-induced collagen deposition and aortic stiffening (117).

Augmented protein O-GlcNAcylation, by inhibiting the OGA enzyme, enhances B cell activation and apoptosis induced by B cell receptor (BCR). B cell-mediated apoptosis occurs through protein O-GlcNAcylation of lymphocyte-specific protein-1 (Lsp1), resulting in protein kinase C-β1-mediated increased levels of Lsp1 phosphorylation with consequent activation of apoptosis-related signaling (153). OGT-deleted mouse exhibit decreased number of mature B increased apoptosis in these cells and defective activation of the B-cell receptor signaling cascade (154), suggesting that O-GlcNAcylation is required for B cell homeostasis and antibody responses since an cells. Inhibition of O-GlcNAc in pre-B cells reduces growth and proliferation due to a decrease in c-Myc expression upon decreased O-GlcNAc, which is important for normal B cell proliferation and cell cycle progression (155). Collectively, these data highlight an important role for protein O-GlcNAcylation in regulating B cells homeostasis.

## Conclusion

Since chronic increases in O-GlcNAcylation levels lead to increased risk of ventricular arrhythmias, myocardial dysfunction, cardiac remodeling, organ damage, aortic banding, and vascular dysfunction, all of them well-known mechanisms whereby blood pressure, it is plays this post-translational modification has been associated with arterial hypertension. On the other hand, recent efforts have been made to determine the role of immune response during high blood pressure and tissue damage in hypertension.

Considering that less than 10% of the patients know the primary cause of their hypertensive disease, one may speculate that the association between O-GlcNAcylation and the activation of the immune system may represent a new clinical approach to the treatment of hypertension. In this sense, current findings in the literature show the many players of innate and adaptive immunity, which are directly involved on vascular inflammation, vascular dysfunction, and hypertension are also targeted by O-GlcNAcylation. As an example, O-GlcNAc-pathway is able to modulate activity, production or pro-inflammatory environment of key component of innate immune response such as, Toll-like receptors, NF-κB pathway, circulating and endothelial cell adhering monocytes, macrophage infiltration and neutrophil lymphocyte ratio, players that are strongly association with the severity of hypertension. Furthermore, it seems clear that O-GlcNAcylation, as well as OGT, play an important role on T and B cell activation, immune cells that are enrolled in the hypertensive disease. Consequently, this post-translational modification modulates IL-2, as well as other cytokines such as IL-4, IFNγ, and TNFα, as observed in activated T cells. Besides, this modification is involved in antibody production, antigen presentation, cytokine production and release through B cell activation. Therefore, the investigation of new potential therapies, specifically aimed to modulate the impact of O-GlcNAc-modified proteins in the innate and adaptive immunity cells to treat or prevent hypertension should be further encouraged.

## Author Contributions

VL, RT, and FG designed the review. RP, GB, FG, and VL wrote the manuscript. VL, FG, and RT revised the manuscript. All authors contributed to the article and approved the submitted version.

## Funding

This work was supported by Fundação de Amparo à Pesquisa do Estado de Mato Grosso (FAPEMAT, 003/2021 to VL); by Conselho Nacional de Desenvolvimento Científico e Tecnológico (CNPq, 141502/2020-7 to RP); the Conselho Nacional de Desenvolvimento Científico e Tecnológico (CNPq, 306166/2019-4 to FG) and São Paulo State Research Foundation (FAPESP; Grant 2013/08216–2 – Research Center in Inflammatory Diseases).

## Conflict of Interest

The authors declare that the research was conducted in the absence of any commercial or financial relationships that could be construed as a potential conflict of interest.

## Publisher’s Note

All claims expressed in this article are solely those of the authors and do not necessarily represent those of their affiliated organizations, or those of the publisher, the editors and the reviewers. Any product that may be evaluated in this article, or claim that may be made by its manufacturer, is not guaranteed or endorsed by the publisher.
